# Respiratory Function and Grip Strength in the Acute Phase of Stroke Are Associated with Stroke Severity and Disability at Hospital Discharge

**DOI:** 10.1155/2020/1636540

**Published:** 2020-02-03

**Authors:** Lorena Cristina Alvarez Sartor, Gustavo José Luvizutto, Juli Thomaz de Souza, Evelin Roberta Silva Dalle Molle, Gabriel Pinheiro Modolo, Taís Regina da Silva, Robson Aparecido Prudente, Priscila Watson Ribeiro, Rafael Dalle Molle da Costa, Letícia Cláudia de Oliveira Antunes, Natália Cristina Ferreira, Silméia Garcia Zanati Bazan, Fernanda Cristina Winckler, Hélio Rubens de Carvalho Nunes, Marcos Ferreira Minicucci, Rodrigo Bazan

**Affiliations:** ^1^PhD Student, Botucatu Medical School, Universidade Estadual Paulista (UNESP), Brazil; ^2^Department of Applied Physical Therapy, Federal University of Triângulo Mineiro (UFTM), Brazil; ^3^Rehabilitation Center, Botucatu Medical School, Universidade Estadual Paulista (UNESP), Brazil; ^4^Stroke Unit, Botucatu Medical School, Universidade Estadual Paulista (UNESP), Brazil; ^5^Department of Internal Medicine, Botucatu Medical School, Universidade Estadual Paulista (UNESP), Brazil; ^6^Statistical, Botucatu Medical School, Universidade Estadual Paulista (UNESP), Brazil; ^7^Department of Neurology, Psychology and Psychiatry, Botucatu Medical School, Universidade Estadual Paulista (UNESP), Brazil

## Abstract

*Introduction*. Stroke can lead to musculoskeletal and respiratory dysfunction, chronic deconditioning, and functional limitations, as well as long-term complications.

**Objective:**

The aim of this study was to evaluate the association between respiratory function and grip strength in the acute phase of stroke and stroke severity, disability, and autonomy in the long term.

**Methods:**

This was a cohort study including 46 patients in the stroke unit. The stroke patients were assessed in the stroke unit at the following moments: at admission by the clinical and haemodynamic stability, demographic and anthropometric data, hand grip strength, stroke severity by National Institutes of Health Stroke Scale (NIHSS) score, and respiratory function using a manovacuometer; during hospitalization by clinical complications and the length of stay; and at hospital discharge and 90 days after discharge by the degrees of functional capacity and dependence using NIHSS, modified Rankin scale (mRs), and Barthel index. Data analysis was performed by multiple linear regression to verify the association between respiratory function and grip strength and the outcomes.

**Results:**

The median length of stay in the stroke unit was 7 days. A negative correlation was found between the palmar prehension strength on the unaffected side and mRs at discharge (*β* = ‐0.034, *p* = 0.049). The NIHSS scores at discharge (*β* = ‐0.034, *p* = 0.049). The NIHSS scores at discharge (*β* = ‐0.034, *p* = 0.049). The NIHSS scores at discharge (

**Conclusion:**

It was concluded that a loss of grip strength is associated with a loss of ability and autonomy at discharge and poor respiratory function is associated with stroke severity at discharge.

## 1. Introduction

Stroke affects approximately 16.9 million people worldwide, and approximately 100,000 people develop functional disability due to stroke per year; stroke is the main cause of mortality and chronic disability in adults in Latin America and Brazil [1, 2]. Stroke is defined as neuronal death caused by prolonged ischaemia due to the obstruction of cerebral blood flow or intracranial haemorrhage [3, 4]. Approximately 90% of patients present with hemiparesis—decreased strength and motor control on one side of the body—after the event, compromising their performance in activities of daily living (ADL), mobility, and locomotion [5, 6].

After stroke, during the period of hospitalization, individuals have a high percentage of muscle loss, muscular weakness, and functional limitations [4]. The main complications during hospitalization include a reduction in chest expansion, respiratory complications, and a loss of muscle strength. Reduced overall physical capacity requires long periods of rehabilitation, which is administered to increase functional capacity and decrease the resulting sequelae of brain injury [7, 8].

The respiratory system can be compromised in the acute phase after stroke. Inactivity due to reduced mobility and low levels of aerobic capacity following stroke results in several dysfunctions, such as reduced cardiorespiratory fitness, a 20% reduction in the cross-sectional area of muscles, and an approximately 25% increase in intramuscular fat, which leads to osteoporosis and circulatory impairment of the lower extremities as well as changes in psychological aspects, such as apathy and depression [9]. In association with the risk of other comorbidities, such as diabetes mellitus, hypertension, and an increased body mass index, the risk of the recurrence of stroke may increase with worsening levels of disability [10].

Previous studies have shown that poststroke weakness, in addition to affecting the upper and lower limb muscles, also affects the inspiratory and expiratory muscles and the kinematic balance of the chest wall. In addition to these skeletal muscle dysfunctions, decreased respiratory function is associated with deconditioning, activity limitations, and respiratory complications; decreased respiratory function is one of the main causes of nonvascular death after stroke [11, 12].

Respiratory complications may also occur due to changes in breathing patterns as well as weakness in the respiratory muscles. Respiratory muscle strength is one of the most important factors in maintaining intact lung function. Respiratory dysfunction results in decreased diaphragmatic movement and chest expansion, increased mechanical resistance to respiration, and decreased ventilation and cough effectiveness, which lead to difficulty in eliminating secretions and significantly increase the individual's risk of lung infections [13, 14].

The main hypothesis of this study is that decreased respiratory function and grip strength are associated with severe cases of stroke, poor functional capacity, and decreased quality of life. The main objective of this study was to evaluate whether respiratory function and grip strength in the acute phase of stroke are related to the severity of stroke, degree of dependence, and physical function at discharge and 90 days after hospital discharge.

## 2. Patients and Methods

This is an observational, prospective study including patients admitted to the stroke unit at Botucatu Medical School. The study was carried out from April 2017 to July 2018. The study was conducted after it was approved by the ethics committee in clinical research at the Botucatu Medical School, and the study number is 1,950,068. Written informed consent was obtained from all subjects before the study.

### 2.1. Inclusion Criteria

Individuals over 18 years of age with a diagnosis of ischaemic stroke that was confirmed by computed tomography (CT) or magnetic resonance imaging (MRI) upon admission and no disability prior to admission, i.e., a score of <1 on the modified Rankin scale (mRs), were included in this study.

### 2.2. Exclusion Criteria

Patients with a history of dysphagia, low scores for items 1a (level of consciousness) and 1c (commands: open/close eyes, tightening and release hands) on the National Institute of Health Stroke Scale (NIHSS), language and facial paresis resulting in a NIHSS score of >1, dementia or other associated neurological diseases, clinical instability, a comatose state, pneumopathy, or chronic valvulopathy and pregnant women were excluded from this study.

### 2.3. Sample Size Calculation

To detect a 2-point difference in the Barthel scale [15] with a statistical power of 0.8 (beta error: 0.2 and alpha error of 0.5), the inclusion of 45 patients was required; the patients were divided into two groups according to the medians of the maximal inspiratory pressure (MIP) and the maximum expiratory pressure (MEP). A total of 46 patients were evaluated, with the target population being patients in the stroke unit; the sample method used was the nonprobabilistic intentional method.

#### 2.3.1. Procedures

The stroke patients were assessed in the stroke unit at the following moments:

- At admission, the clinical and haemodynamic stability, demographic and anthropometric data, hand grip strength, NIHSS score, and respiratory function were assessed
- During hospitalization, clinical complications were assessed, and the length of stay was recorded
- At discharge and 90 days after discharge, the degrees of functional capacity and dependence were also evaluated by specific scales

#### 2.3.2. Evaluation Tools

The following clinical and haemodynamic variables were assessed:

- Respiratory frequency (RF): RF was determined by counting the respiratory incursions that occurred over 60 seconds
- Blood pressure (BP): BP was measured directly from the Dixtal® monitor with a sphygmomanometer coupled to the unaffected arm of the patient
- Heart rate (HR): HR was measured directly from the Dixtal® monitor by adhesive electrodes attached to the patient's chest
- Peripheral oxygen saturation (SPO2): SPO2 was measured by a pulse oximeter coupled to the third metacarpal on the unaffected side of the patient and transmitted to the monitor

### 2.4. Anthropometric Variables

Body weight (kg) was measured using a Filizola® digital scale for patients who could stand up and an electronic balance attached to a crane for the bedridden patients. Stature (m) was measured by a stadiometer fixed to the wall for the patients who were able to stand. When it was not possible to measure a patient's weight and height, these parameters were estimated by the nutritionist responsible for the stroke unit by formulas considering the patient's age, race, arm circumference, and knee height [16, 17]. After the weight and height were obtained, the body mass index (BMI) was calculated using the Quetelet formula:BMI (kg/m^2^) = weight (kg)/height^2^ (m) [18].

### 2.5. Grip Strength Evaluation

To assess grip strength, the manual gripping force was measured. Each individual was positioned in a chair without support, with the hip and knees flexed to 90 degrees and with the feet rested on the floor. The upper limb to be evaluated was positioned with the shoulder in the adducted position; the elbow flexed to 90 degrees; the forearm in the neutral position, which was between 0 and 30 degrees of extension; and the wrist in 0 to 15 degrees of adduction. The limb that was not tested was placed on the ipsilateral thigh. The participant was asked to grip the hand with maximum force for 3 seconds, and a rest interval of 30 seconds was provided between the tests; the average values from the three tests were calculated for each hand. The handgrip strength was evaluated on the affected and nonaffected sides. All participants were asked to maintain their posture throughout the test, and their posture was corrected when necessary by the evaluator [19].

### 2.6. Risk Factors

The risk factors were recorded by a neurologist using anamnesis when the patient was admitted to the hospital. The following risk factors were evaluated: systemic arterial hypertension (AHT), smoking habit, obesity, alcoholism, Chagas' disease, congestive heart failure (CHF), coronary artery disease (CAD), diabetes, dyslipidaemia, depression, stroke or a history of transient ischaemic attack (TIA), and a history of acute myocardial infarction (AMI).

### 2.7. Respiratory Function

Respiratory function was evaluated by respiratory muscle strength using a manovacuometer with an operating range of ±120 cm H_2_O (Support®, São Paulo, Brazil). The maximal inspiratory pressure (MIP) and the maximum expiratory pressure (MEP) were measured using the Black and Hyatt method, considering the predicted maximum respiratory pressure reference values corresponding to the patient's age and sex [20, 21].

- Maximum inspiratory pressure (MIP): the MIP was measured with the patient in bed positioned at 45°; the patient was to exhale with maximum effort until the residual volume level was reached, to inhale with maximum effort against the occluded airway, and to sustain the inhale for one second. The pressure value was directly displayed on the manovacuometer. The manoeuvres were repeated three times, with intervals of one minute between evaluations, and the highest value was used for analysis
- Maximum expiratory pressure (MEP): to evaluate the MEP, the patient was also placed at 45° in the bed and asked to inhale with maximum effort until the total lung capacity was reached. The patient was then asked to exhale with maximum effort against the occluded airway, which was sustained for one second. The pressure value was directly displayed on the manovacuometer display. The manoeuvres were repeated three times in intervals of one minute, and the highest value was used for analysis

During the execution of the MIP and MEP manoeuvres, the patients used a nasal clamp and a mouthpiece with a small hole that prevents closure of the glottis with the inspiratory flow during the manual occlusion of the manovacuometer [21].

### 2.8. Degree of Dependency

To determine the degree of dependency, the Barthel index was used, which is an instrument used to evaluate the degree of dependence of an individual for 10 basic activities of daily living (ADL). The scale has a minimum score of 0 and a maximum of 100 points; higher scores correspond to higher degrees of independence and better performance in the execution of each activity [22].

### 2.9. Functional Capacity

The modified Rankin scale (mRs) was used to evaluate the functional capacity of the individual. The scale is an ordinal scale from 0 to 6, and a lower score indicates less impairment; a larger score indicates a worse outcome. A score of 6 indicates death [23].

### 2.10. Severity of Neurological Deficits

The severity of neurological deficits was verified by the NIHSS, which is an instrument used for the quantitative evaluation of neurological deficits; the reliability and validity of the instrument have been established for use in clinical research. It is composed of 11 items divided into the following domains: consciousness level (1a, 1b, and 1c), eye movements (2), integrity of visual fields (3), facial paralysis (4), right and left arm motor function (6), limb ataxia (7), sensation (8), language (9), dysarthria (10) and neglect or inattention (11). Each item is scored on a scale ranging from 0 to 2, 0-3, or 0-4, in addition to nontestable items. The total score may reach values of 0-42 points; the higher the NIHSS value, the more severe the case of stroke. The scale was administered by both medical staff and health professionals with proven training and certification [23].

### 2.11. Statistical Analysis

Data are presented as the mean ± standard deviation, median, and 25 and 75% percentiles or percentage. Continuous variables were analysed by Student's *t*-test (when their distribution was normal) or by the Mann–Whitney test (when the distribution was nonnormal). To evaluate the association between two continuous variables, the Spearman correlation test was used. Multiple linear regression was performed for the following outcomes at discharge and 90 days: mRs, Barthel index, and NIHSS score. These dependent variables were normalized when they had a nonnormal distribution. The adjustments made in the multiple regression models were based on clinically relevant variables according to the literature. Data analysis was performed using SigmaPlot software for Windows v12.0 (Systat Software Inc., San Jose, CA, USA). The level of significance was 5%.

## 3. Results

The results of the demographic and clinical data of the included patients are presented in Table 1. Of the patients evaluated, all underwent rehabilitation treatment (physiotherapy, occupational therapy, and/or speech therapy) during hospitalization in the stroke unit. Regarding the clinical and haemodynamic variables, the values obtained within 72 hours were within the normal range, which allowed manovacuometry to be performed and the health of the individuals to be preserved during and after evaluation.

During hospitalization, the patients presented some complications; the complication with the highest incidence was decompensated arterial pressure (8.7%) (4), followed by headache (8.7%) (4), hypotension (4.3%) (2), and dyspnoea (4.3%) (2). After discharge, a total of 34.8% (16) of the individuals had one or more complications; however, statistically significant associations with the variables of this study were not found.

Table 2 shows the results of the Spearman correlation test, which verified the relation of the independent variables with mRs. A correlation was found for the NIHSS score at admission (*p* = 0.013), the NIHSS score at discharge (*p* < 0.001), and handgrip strength on the affected side (*p* = 0.010) with the mRs at discharge. For the mRs at 90 days after discharge, a correlation was found with the NIHSS score at admission (*p* = 0.050), the NIHSS score at discharge (*p* < 0.001), SBP (*p* = 0.035), and palmar prehension strength on the affected side (*p* = 0.006). These variables were used in multiple linear regression analysis, and confounding factors (stroke severity—NIHSS score at admission, sex, and age) were considered. In the multiple linear regression analysis (Table 3), a negative correlation was found between the palmar prehension strength on the unaffected side and mRs at discharge (*β* = ‐0.034, *p* = 0.049) after it was corrected by the patient's age, sex, and NIHSS score at admission.

Table 4 shows that the NIHSS score at admission had a negative correlation with palmar prehension strength on the affected side (*p* = 0.023). The NIHSS score at 90 days after discharge showed a negative correlation with systolic blood pressure (SBP) (*p* = 0.030) and palmar prehension strength on the affected side (*p* = 0.002) according to the Spearman correlation test. In the multiple linear regression analysis (Table 5), only the NIHSS score at discharge showed a negative correlation with the MEP (*β* = ‐0.016, *p* = 0.006) after the model was corrected for the patient's age, sex, and NIHSS score at admission.

Table 6 shows that the Barthel scale at hospital discharge had a negative correlation with the NIHSS score at discharge (*p* < 0.001). In the multiple linear regression analysis, the Barthel index at discharge had a positive correlation with the palmar prehension strength on the unaffected side (*β* = 0.480, *p* = 0.023) after the model was corrected for the patient's age, sex, and NIHSS score at admission (Table 7).

## 4. Discussion

In this study, we found a negative correlation between the mRs at discharge and handgrip strength on the unaffected side, a negative correlation between the severity of stroke measured by the NIHSS and the MEP, and a positive correlation between the Barthel index at discharge and the palmar prehension strength on the unaffected side.

The NIHSS score is the most widely used severity stroke scale, and it can predict survival, functional recovery, and patient's postacute care disposition [8, 23]. Hence, measures of dependence (mRs and Barthel) are widely used disability and autonomy in stroke trials [8, 23]. The main findings of this study confirm the main hypothesis that there is an association between respiratory function and grip strength in the acute phase of stroke and the functional capacity, degree of dependence, and severity of stroke in the long term.

The manual gripping force can also be an indicator of long-term functionality due to its association with mRs at discharge. There was a negative correlation between hand grip strength in the acute phase of stroke and the mRs at hospital discharge adjusted for stroke severity, age, and sex in the present study. Longitudinal studies in large cohorts have demonstrated that a decrease in manual grip strength is associated with an increase in the number of hospitalizations, morbidity, and mortality [24]. In individuals with stroke, lower palmar grip strength is associated with lower activation of the primary motor cortex and is responsible for muscle strength control, which may explain the lower functionality and larger degree of dependence of these individuals [25].

Individuals presenting with more severe cases of stroke have greater motor impairment and reduced muscle activity, which may explain the relationship between stroke severity and the strength of the lower expiratory musculature. The NIHSS score is representative of the overall severity of the individual's neurological impairment after stroke, and higher scores correlate with worse neurological impairment, lower functional capacity, a larger injured area, and greater dependence in daily life activities [26, 27]. A more severe neurological condition, as measured by the NIHSS score, was associated with a decline in the MEP. Individuals presenting with more severe conditions tend to show a decline in motor functioning, and those with higher NIHSS scores can show reduced physical conditioning and reduced strength in expiratory function. Expiratory muscle strength is related to abdominal muscle function, and previous studies have reported decreased respiratory forces in individuals during the acute and chronic stages after stroke [28, 29]. The abdominal muscles are responsible for stabilizing the trunk during the main functional activities and are important for sitting, standing, and ambulation. The weakness of these muscles, assessed by the maximum expiratory pressure, can represent a loss of trunk muscle function, a loss of postural control, and dependence during ADL [30, 31].

In this study, we found a positive correlation between the Barthel index at discharge and palmar prehension strength on the unaffected side; therefore, the stronger the palmar grip, the more independent the individual is. Muscle strength is functionally very important in daily life, and this aspect is usually evaluated in rehabilitation. Manual grip strength is affected by several factors, but in an individual with stroke, it is highly related to hemiparesis; individuals with worse neurological status may present with worse muscle strength [32, 33]. Palmar grip strength is an indicator of overall muscle strength, and it is important for the individual to have autonomy and perform activities of daily living independently.

This study has some limitations. The population in a single stroke unit is always selective and is limited in number, but we consider the sample size adequate for the purposes of this study. Our study population consisted mainly of individuals with mild and moderate stroke due to the importance of the patient being conscious and cooperative during the assessment. The conventional instruments used in this study (NIHSS, mRs, and Barthel) do not measure the quality of the individual's performance in activities of daily living after stroke. The NIHSS score is not directly associated with an individual's ability to compensate for a neurological deficit, and the mRs and Barthel index (BI) do not include questions about body functions, activities, or participation. Individuals may have varying degrees of functional recovery within 90 days, depending on the activity they performed and the environmental context in which they were living. Therefore, we suggest using a functional model for evaluating recovery after stroke.

## 5. Conclusion

Based on the results, it was concluded that decreased grip strength function is associated with higher disability and lower autonomy at discharge, and poor respiratory function in acute phase of stroke is associated with stroke severity at hospital discharge.

## Figures and Tables

**Table 1:** Demographic and clinical variables of the included patients (*n* = 46).

Variables	*N*	%
Demography		
Male	30	65.2
Age (years)	62.9 (42-76)	
Race		
Caucasian	37	80.4
Non-Caucasian	9	19.6
Risk factors		
Hypertension	35	76.1
Smoking habit	24	52.2
Obesity	13	28.3
Diabetes	9	19.6
Drinking habit	9	19.6
Atrial fibrillation	6	13
Hypothyroidism	6	13
Depression	5	10.9
Dyslipidaemia	4	8.69
Valvulopathy	4	8.69
CAD	3	6.52
Myocardial infarction (previous)	2	4.35
Sleep apnoea syndrome	1	2.17
Congestive heart failure	1	2.17
BAMFORD		
LACS	28	60.9
PACS	11	23.9
POCS	6	13.1
TACS	1	2.1
TOAST		
Indeterminate	27	58.7
Cardioembolic	9	19.6
Small vessel disease	7	15.2
Large vessel disease	3	6.5
Other causes	0	
Haemodynamic variables		
Blood pressure (mmHg)		
Systolic (mmHg)	142.8 (99-193)	
Diastolic (mmHg)	83.8 (55-177)	
Mean (mmHg)	105.7 (84-157)	
Heart rate (bpm)	71.2 (50-104)	
Respiratory rate (rpm)	18.8 (16-21)	
Saturation of peripheral oxygen (%)	96.1 (92-100)	
Rankin prestroke		
0	37	80.4
1	9	19.6
NHISS score at admission^1^	3.4 (0-10)	
Glycaemia (mg/dl)	121.1 (58-377)	
Thrombolysis	8	17.4
Length of stay at hospital	7 (3-15)	

AF: atrial fibrillation; TIA: transient ischaemic attack; CHF: congestive heart failure; CAD: coronary artery disease; LACS: lacunar syndrome; TACS: total anterior circulation syndrome; PACS: partial anterior circulation syndrome; POCS: posterior circulation syndrome; NIHSS: National Institute of Health Stroke Scale. The results are expressed as the median and percentiles or percentage.

**Table 2:** Clinical and demographic variables, respiratory function, and grip strength correlation with the modified Rankin scale score.

Variables	mRs at discharge		mRs 90 days after discharge	
	*r*	*p*	*r*	*p*
Age (years)	0.070	0.662	-0.030	0.848
NIHSS at admission	0.370	**0.013**	0.300	**0.050**
NIHSS at discharge	0.670	**<0.001**	0.600	**<0.001**
Glycaemia	0.134	0.373	0.040	0.787
Weight	0.090	0.567	-0.153	0.308
Height	-0.030	0.865	-0.073	0.628
BMI	0.124	0.411	0.015	0.922
AC	0.080	0.614	-0.105	0.484
RR	-0.200	0.189	-0.108	0.475
CR	-0.020	0.908	-0.113	0.455
SPO2	0.020	0.899	0.173	0.249
SBP	-0.090	0.525	-0.311	**0.035**
DBP	-0.230	0.126	-0.213	0.154
MBP	-0.130	0.393	-0.263	0.077
MIP	-0.190	0.206	-0.098	0.513
MEP	-0.250	0.098	-0.238	0.110
Handgrip affected side	-0.376	**0.010**	-0.397	**0.006**
Handgrip nonaffected side	-0.169	0.260	-0.043	0.772
Hospital stay	0.090	0.571	0.090	0.548

mRs: modified Rankin scale; *r*: correlation coefficient; NIHSS: National Institutes of Health Stroke scale; BMI: body mass index; AC: abdominal circumference; RR: respiratory rate; HR: heart rate; SPO2: saturation of peripheral oxygen; SBP: systolic blood pressure; DBP: diastolic blood pressure; MBP: median blood pressure; MIP: maximum inspiratory pressure; MEP: maximum expiratory.

**Table 3:** Association between respiratory function and grip strength and functional disability, as measured by modified Rankin scale, at discharge and 90 days after discharge.

mRs at discharge			
Variables	*β*	SE	*p*
MEP^∗^	-0.007	0.004	0.104
MEP^∗∗^	-0.009	0.005	0.062
MIP^∗^	-0.005	0.004	0.228
MIP^∗∗^	-0.005	0.005	0.295
Handgrip nonaffected side^∗^	-0.020	0.013	0.128
Handgrip nonaffected side^∗∗^	**-0.034**	**0.017**	**0.049**

mRs 90 days after discharge			
Variables	*β*	SE	P
MEP^∗^	-0.005	0.003	0.111
MEP^∗∗^	-0.007	0.004	0.057
MIP^∗^	-0.002	0.003	0.534
MIP^∗∗^	-0.004	0.003	0.188
Handgrip nonaffected side^∗^	-0.002	0.010	0.881
Handgrip nonaffected side^∗∗^	-0.012	0.013	0.365

mRs: modified Rankin scale; MIP: maximum inspiratory pressure; MEP: maximum expiratory pressure; *β*: beta error; SE: standard error. ^∗^Adjusted for the NIHSS score at admission. ^∗∗^ Adjusted for the age, sex and NIHSS score at admission.

**Table 4:** Association between the demographic, clinical, respiratory function, and grip strength variables and stroke severity, as measured by the NIHSS scale.

Variables	NIHSS score at discharge		NIHSS score 90 days after discharge	
	*r*	*p*	*r*	*p*
Age (years)	-0.063	0.677	-0.117	0.436
Glycaemia	-0.019	0.899	-0.066	0.661
Weight	-0.029	0.843	-0.137	0.363
Height	-0.0004	0.997	-0.151	0.316
BMI	-0.037	0.804	0.027	0.857
AC	-0.167	0.265	-0.117	0.436
RR	-0.056	0.710	-0.058	0.698
CR	0.014	0.928	-0.163	0.279
SPO2	0.076	0.614	0.073	0.629
SBP	-0.037	0.807	-0.315	**0.030**
DBP	-0.010	0.946	-0.256	0.086
MBP	0.032	0.831	-0.228	0.127
MIP	-0.273	0.066	-0.066	0.662
MEP	-0.271	0.068	-0.162	0.281
Handgrip strength affected side	-0.335	**0.023**	-0.450	**0.002**
Handgrip strength nonaffected side	-0.126	0.404	-0.067	0.658
Length of hospital stay	0.194	0.195	0.076	0.614

*r*: Correlation coefficient; NIHSS: National Institutes of Health Stroke Scale; BMI: body mass index; AC: abdominal circumference; RR: respiratory rate; HR: heart rate; SPO2: saturation of peripheral oxygen; SBP: systolic blood pressure; DBP: diastolic blood pressure; MBP: median blood pressure; MIP: maximum inspiratory pressure; MEP: maximum expiratory pressure.

**Table 5:** Association between respiratory function and grip strength and the severity of stroke (NIHSS score) at discharge and 90 days after discharge.

NIHSS at discharge			
Variables	*β*	SE	*p*
MEP^∗^	-0.102	0.005	0.059
MEP^∗∗^	**-0.016**	**0.006**	**0.011**
MIP^∗^	-0.008	0.005	0.147
MIP^∗∗^	-0.010	0.006	0.075
Handgrip nonaffected side^∗^	-0.015	0.016	0.364
Handgrip nonaffected side^∗∗^	-0.040	0.021	0.057

NIHSS 90 days after discharge			
Variables	*β*	SE	*p*
MEP^∗^	-0.004	0.004	0.302
MEP^∗∗^	-0.007	0.004	0.119
MIP^∗^	-0.001	0.004	0.688
MIP^∗∗^	-0.004	0.004	0.375
Handgrip nonaffected side^∗^	-0.009	0.011	0.407
Handgrip nonaffected side^∗∗^	-0.027	0.014	0.063

mRs: modified Rankin scale; MIP: maximum inspiratory pressure; MEP: maximum expiratory pressure. Multiple linear regression tests were used (*p* < 0.05); *β*: beta error; SE: standard error. ^∗^Adjusted for NIHSS score at admission. ^∗∗^Adjusted for the age, sex, and NIHSS score at admission.

**Table 6:** Association between the demographic, clinical, respiratory function, and grip strength variables and the degree of dependence, as measured by the Barthel index.

Variable	Barthel at discharge		Barthel 90 days after discharge	
	*r*	*p*	*r*	*p*
Age (years)	-0.204	0.172	-0.179	0.233
NIHSS at admission	-0.218	0.144	-0.137	0.363
NIHSS at discharge	-0.483	**<0.001**	-0.217	0.148
Glycaemia	0.031	0.836	-0.035	0.815
Weight	-0.085	0.569	0.250	0.093
Height	-0.163	0.278	-0.140	0.353
BMI	0.022	0.883	0.278	0.062
AC	0.119	0.429	0.234	0.177
RR	0.025	0.869	0.095	0.259
CR	-0.064	0.667	-0.059	0.694
SPO2	-0.215	0.150	-0.218	0.145
SBP	0.127	0.400	0.114	0.447
DBP	-0.027	0.857	0.186	0.215
MBP	-0.017	0.911	0.126	0.401
MIP	0.027	0.855	0.178	0.235
MEP	0.019	0.899	0.138	0.358
Handgrip affected side	0.103	0.493	0.094	0.533
Handgrip nonaffected side	0.025	0.870	0.002	0.986
Hospital stay	-0.045	0.768	0.059	0.693

*r*: correlation coefficient; NIHSS: National Institutes of Health Stroke scale; BMI: body mass index; AC: abdominal circumference; RR: respiratory rate; HR: heart rate; SPO2: saturation of peripheral oxygen; SBP: systolic blood pressure; DBP: diastolic blood pressure; MBP: median blood pressure.

**Table 7:** Association between respiratory function and grip strength and the degree of dependence, as measured by the Barthel index, at discharge and 90 days after discharge.

Barthel index at discharge			
Variables	*β*	SE	*p*
MEP^∗^	0.027	0.059	0.649
MEP^∗∗^	0.071	0.062	0.256
MIP^∗^	0.043	0.058	0.462
MIP^∗∗^	0.036	0.059	0.549
Handgrip strength nonaffected side^∗^	0.184	0.171	0.287
Handgrip strength nonaffected side^∗∗^	**0.480**	**0.203**	**0.023**

Barthel index 90 days after discharge			
Variables	*β*	SE	*p*
MEP^∗^	0.016	0.018	0.395
MEP^∗∗^	0.033	0.020	0.103
MIP^∗^	0.012	0.018	0.499
MIP^∗∗^	0.018	0.019	0.342
Handgrip strength nonaffected side^∗^	0.017	0.054	0.752
Handgrip strength nonaffected side^∗∗^	-0.043	0.053	0.432

MIP: maximum inspiratory pressure; MEP: maximum expiratory pressure; multiple linear regression test was used (*p* < 0.05); *β*: beta error; SE: standard error. ^∗^Adjusted for NIHSS score at admission. ^∗∗^Adjusted for the age, sex, and NIHSS score at admission.

## Data Availability

The table data with patients details (excel) used to support the findings of this study are available from the corresponding author upon request.
